# The Role of Vitamins and Minerals in Psychiatry

**Published:** 2008-09-24

**Authors:** Stacey Cornish, Lewis Mehl-Madrona

**Affiliations:** University of Saskatchewan College of Medicine

## Introduction

Roughly 90 years of research demonstrate the relevance of dietary nutrients for mental health. Some of the earliest research studies on nutrients relevant to mental illness observed irritability and mood problems in people known to be deficient in the B vitamins^1^, as well as reported positive improvements in mental illness when treated with such nutrients as manganese^2^,^3^ and nicotinic acid;^4^ regardless of whether or not the patients could be found to be deficient. Although interest in such studies have declined since the introduction of psychiatric medications in the 1950’s, recent work on folic acid (vitamin B9) suggests that low levels may be associated with depressive symptomatology and poor response to antidepressant medication.^5^

Increasing evidence about the effects of trace elements on brain and behavioral functioning is appearing as well. Zinc, copper, and magnesium may play an important modulatory role in controlling a subtype of glutamate receptor (NMDA receptor),^6^ glutamate being the primary transmitter for most excitatory neurons in the cerebral cortex. This NMDA receptor has been implicated in various forms of cortical functioning;^7^ therefore it appears that decreased levels of these nutrients may produce abnormal NMDA activity and subsequent abnormal behavior. Given the accumulating evidence from PET and fMRI imaging studies showing that schizophrenia and affective disorders are associated with abnormal cortical activity,^8^ it is logical to state that such conditions could result, at least in part, from abnormalities in the nutritional status of neurons. Other studies regarding the relevance of nutrients and schizophrenia have been conducted as well. Comparison studies have shown that 26 medication-free schizophrenics were found to have significantly low serum iron,^9^ in addition to a study in Israel where both the cerebrospinal fluid and serum of people with schizophrenia were tested to be low in magnesium.^10^ Still others have studied essential fatty acid-related membrane processes. Among 38 schizophrenics and 22 controls, in the cutaneous flushing response to aqueous methyl nicotinate,: 83% of the people with schizophrenia (but only 23% of the controls) exhibited the absence of a flushing response, indicative of deficient levels of arachidonic acid.^11^ This particular study is relevant due to the fact that some minerals (e.g. Zinc) are thought to be rate-limiting factors in essential fatty acid conversion pathways.^12^

High dose vitamin therapy has been studied with a number of genetic diseases. The molecular basis of disease arising from as many as one-third of the mutations in a gene is an increased Michaelis constant, or K_M_ (decreasing binding affinity) of an enzyme for the vitamin-derived coenzyme or substrate, which in turn lowers the rate of the reaction.^13^ The K_M_ is defined as the concentration of ligand required to fill one-half of the ligand binding sites. Therapeutic vitamin regimens are thought to increase intracellular (cofactor) concentration, thus activating a defective enzyme, which alleviates the primary defect and remediates the disease. The proportion of mutations in a disease gene that is responsive to high concentrations of a vitamin or substrate may be one-third or greater.^14^,^15^,^16^

The battle to reduce the stigma associated with nutritional therapies is still very present today, fifty years later. More commonly labeled “alternative medicine/therapy,” nutritional therapies are considered just that: an alternative, a last resort, or are not considered at all. Over the years, major medical textbooks have claimed that “routine prescription of vitamin preparations is indefensible, it is poor medical practice,^17^ and that “multivitamins are not necessary.^18^ Goodwin went so far as to say that a bias exists in this particular area; where “positive results are viewed with suspicion,” and “negative results are published in the best journals.”^19^ One study found that most doctors do not feel comfortable discussing alternative therapies with their patients, despite the fact that 55% of patients have requested more information about herbal (or natural) medicine.^20^ However, despite the presence of skeptics, criticisms and lack of information to the public, natural therapies continue to be used.

We will review a number of substances and their potential use in psychiatry.

### Essential fatty acids

Essential Fatty Acids (EFA) must be obtained either from diet or through supplementation. Premenstrual Dysphoric Disorder (PMDD) affects many women in varying degrees, ranging from only physical symptoms before the menses to varying degrees of irritability, anger, and depression. Ranging from mild to severe, PMDD can be treated with Evening Primrose Oil (EPO). EPO contains two essential precursors for prostaglandin synthesis, 70% cis-linoleic acid, and 8 to 14% gamma-linolenic acid. By providing the body with these essential fatty acids, EPO facilitates the synthesis of Prostaglandin E_1_ (PGE_1_); a substance that women with PMDD may lack in the central nervous system as well as in other tissue including the breast tissue.^21^

In an open trial, 18 women with PMDD of more than 1 year’s duration received 8 capsules/day of evening primrose oil in the last half of the menstrual cycles for 5 cycles.^22^ Irritability (*p* < 0.001), depression (*p* < 0.001), anxiety (*p* < 0.01), and fatigue (*p* < 0.01) were significantly less compared to baseline after the first cycle of treatment. Total PMS scores were significantly improved (*p* < 0.001).

A Cochran Database review concludes that limited evidence gives support to a hypothesis suggesting that the symptoms of schizophrenia may result from altered neuronal membrane structure and metabolism.^23^ The latter are dependent on blood plasma levels of certain essential fatty acids (EFAs) and their metabolites. They found several studies showing that those with schizophrenia often have low levels of the particular EFAs necessary for normal nerve cell membrane metabolism. Four relatively small trials (total n = 204) showed low levels of loss to follow up and adverse effects for those taking essential fatty acids. Early results from a few trials suggest a positive effect of eicosapentaenoic acid (EPA) over placebo for scale-derived mental state outcomes. The data, however, was limited, making these results difficult to analyze and interpret with confidence. A single small study (n = 30) investigated the value of using EPA as sole treatment for people hospitalized for relapse. Results suggested that EPA could help one third of people avoid instigation of standard antipsychotic drugs for 12 weeks (RR 0.6, CI 0.4–0.91). There were no clear effects of primrose oil (omega-6) EFA supplementation. Omega-3-Fatty acids have been used as treatment for depression,^24^ especially eicosapentaenoic acid.^25^,^26^,^27^

## Vitamin B6 and Magnesium

For forty years, vitamin B6 has been used in various amounts to treat people with autism, primarily younger children. Several studies have shown that many children with autism present with low B6, both the activated form (42% in one study)^28^ as well as the functional form. Some of the earliest studies include an open trial in which twelve of sixteen improved after treatment with B6, three of them speaking for the very first time.^29^ In 1978, Rimland, Callaway and Dreyfus conducted a double-blind placebo-controlled trial in which eleven of fifteen children were classified as better on B6.^30^

In a survey of 4000 parents of autistic children, high dose pyridoxine and magnesium treatment (n = 318) elicited the best response.^31^ For every parent reporting behavioral worsening with this treatment, 8.5 parents reported behavioral improvement. The next best results were with the acetylcholine precursor, deanol (n = 121) with 1.8 parents reporting a favorable response compared to 1 reporting a negative response. Sixteen autistic children previously responsive to pyridoxine treatment were reassessed and randomized to pyridoxine or placebo. Behavior deteriorated significantly during pyridoxine withdrawal and 11 of 15 children behaved better when given 300 mg/day of pyridoxine.^32^ A double blind trial involving 60 autistic children found that 30 mg pyridoxine/kg/day up to doses of 1000 mg/day and magnesium, 10–15 mg/kg/day, were more helpful than either supplement alone in relieving the symptoms of autism alone. Patients receiving combined treatment showed a significant (p = 0.02) decrease in homovanillic acid excretion and significant clinical improvement.^33^ A dozen other reports (with up to 190 participants) since 1965 and a review of clinical trials^34^ have reported improvements in autistic children with pyridoxine and often magnesium supplements,^35^ although the conclusions have been challenged.^36^

Within the past five years more and more studies have been conducted that include magnesium as part of their treatment regimen. An extensive study done by Rimland and Edelson of 5780 autistic patients (both children and adults) was done where varying doses of both B6 and Magnesium were given. This treatment was found to show improvement in 47% of its subjects.^37^.

## Thiamine (Vitamin B1)

For anxiety, thiamine has been used successfully at doses of 250 mg/day to treat patients with anxiety disorders, including symptoms manifesting as chronic fatigue, insomnia, nightmares, anorexia, nausea and vomiting, diarrhea or constipation, chest and abdominal pain, depression, aggression, headache, diaphoresis, and fevers of unknown origin. Among over 200 subjects, successful responders had deficient RBC transketolase which normalized in 73% of the subjects and led to disappearance or great clinical improvement in most of the symptoms.^38^

The National Academy of Sciences (U.S.) has not set a tolerable upper level for thiamine, stating that no adverse effects have been reported for thiamine. The DRI’s for thiamine for men and women are 1.2 and 1.1 mg/day, respectively.^39^ Thiamine is phosphorylated to form TPP, the cofactor used by many enzymes.

Twenty-six patients with Leigh’s disease responded to high intakes of thiamin, doses ranging from 20 to 3000 mg/day. In two sisters, lipoic acid (100 mg/day) plus thiamine (3000 mg/day) provided the best remediation. Leigh’s disease is caused by genetic defects in the pyruvate dehydrogenase multienzyme complex (PDHC) that uses TPP, lipoic acid, CoA, FAD, and NADH coenzymes to catalyze the conversion of pyruvate to acetyl-CoA. The gene encoding the E1alpha peptide of the E1 subunit (pyruvate decarboxylase), which binds TPP, is located on the X-chromosome. Leigh’s disease is associated with lethal lactic acidosis, psychomotor retardation, central nervous system damage, ataxia, muscle fiber atrophy, and developmental delay.^40^ Pyruvate and lactate accumulate with resulting encephalomyopathy. Enzymes from responsive patients show a reduced affinity for TPP.^41^

## Vitamin B6 (Pyridoxine)

One hundred and eighty-nine subjects with either generalized anxiety disorder, panic disorder, or obsessive-compulsive disorder were evaluated for plasma pyridoxal phosphate (PLP) levels and compared with normal controls. There was no difference in plasma PLP levels between the anxiety disorder groups and normal controls. Low levels of plasma PLP were found in 42% of the controls. The results suggested that previous reports of low PLP levels in psychiatric patients were unlikely to be significant in the etiology of the psychiatric disorders.^42^ Critics of this study point out that blood levels vary last in most medical conditions and do not necessarily reflect tissue levels or levels within the central nervous system. Blood levels of neurotransmitters are also typically normal in psychiatric patients. Vitamin B6 and folic acid.^43^,^44^,^45^,^46^,^47^,^48^ have been used successfully for depression. Inositol is, unofficially, a B vitamin that is found in cell membranes (phosphatidylinositol) where it functions closely with choline.^49^ Inositol was equivalent to single-drug therapy for depression and panic disorder in one study and is a potential antidepressant.^50^,^51^ One study found 75% of depressed patients to be magnesium deficient with another 9% at borderline levels.^52^ Magnesium supplementation was helpful.^53^

The Institute of Medicine has established an upper tolerable limit of 100 mg/day for adults for vitamin B6 (pyridoxine). The recommended daily intake is 1.3 mg/day for adults. In the liver, pyridoxine and pyridoxal (an oxidized form) are phosphorylated by pyridoxal kinase to form pyridoxine-P and PLP, the active cofactor form. Pyridoxine-P is oxidized to PLP by pyridoxine oxidase. PLP is used by 112 (3%) of the 3870 catalogued enzymes in the ENZYME database.^54^ The cofactor forms a covalent linkage (Schiff base) with a lysyl residue in the enzyme. This internal aldimine (enzyme-PLP) is converted to an external aldimine (substrate-PLP) when PLP is attacked by a substrate amino group. Gyrate atrophy of the choriod and the retina is an autosomal recessive disease leading to blindness and affecting all ages. It is caused by defects in ornithine aminotransferase (OAT), a PLP-dependent mitochondrial matrix protein that catalyzes the breakdown of ornithine to delta-pyrroline-5-carboxylic acid, which is then converted to proline. The disease is characterized by slowly progressive chorioretinal degeneration leading to blindness. Ornithine accumulates 10 to 15 fold when the enzyme is defective and appears to be responsible for much of the gyrate atrophy.^55^ A pyridoxine responsive form of the disease exists in which high doses of pyridoxine (10 to 750 mg/day) lead to decreased ornithine accumulation and reduced disease severity.

The OAT activity in fibroblast extracts of a pyridoxine-responsive patient with the alanine-to-valine substitution at codon 222 (Ala226 → Val) increased from 9 to 44 nmol product/mg/h when the concentration of PLP in the assay was increased to 600 micromoles/L.^56^ Vitamin B6 responsive and non-responsive patients were shown to have different point mutations resulting in a single amino acid change in the mature enzyme.^57^ OAT activity increased substantially more after incubation with PLP in responsive patients compared to non-responsive patients. In another study, three patients responded to oral vitamin B6 (600 to 750 mg/day) with a decrease in serum ornithine and a return to normal of reduced concentrations of serum lysine. Lower doses of vitamin B6 (18–30 mg/day) appeared to work as well as high doses.^58^ In another study of 9 patients with gyrate atrophy, four responded to pyridoxine with greater than 50% reductions in ornithine levels. The non-responders were thought to harbor more severe genetic mutations. In a Japanese study of 9 patients, only one responded to pyridoxine, and that individual had a Thr181 → Met mutation.^59^ In another Japanese study, one of three patients responded to pyridoxine (300–600mg/day) with a 60% reduction in serum ornithine concentrations. A Glu318 → Lys mutation of the OAT gene was found in three heterozygous pyridoxine responders and one homozygous responder. No adverse effects of pyridoxine treatment at doses up to 750 mg/day were reported.

A double blind placebo controlled crossover study found high doses of pyridoxine (<= 400 mg/day) to be effective in reducing symptoms of tardive dyskinesia in patients with schizophrenia.^60^ (Pyridoxine was added to the normal neuroleptic treatment of all 15 patients in the study for 4 weeks at a time, split by a one week washout period. Pyridoxine treatment resulted in improvements in both the dyskinetic movement and Parkinsonian subscales with return to baseline when pyridoxine was withdrawn An earlier pilot study by the same group showed significant clinical improvement in 4 of 5 tardive dyskinesia patients given 100 mg/day pyridoxine on top of their usual treatment.^61^ Three of the responders also showed significant improvement on the Brief Psychiatric Rating Scale.

The double-blind study just mentioned showed that baseline plasma PLP levels could be raised from 49 nmol/L to 690 nmol/L (a more than 14 fold increase) safely with a dose of 400 mg per day of pyridoxine. A rate study showed that extremely large doses are well absorbed and tolerated.^62^ Although dosages in the hundreds of milligrams have been safely applied, reports exist of neurotoxic effects with very high pyridoxine use. One review advises avoiding doses greater than 1000 mg per day.^63^ The Merck Manual reports toxicity effects at 2000 mg/day and suggest a safe level of 200 mg/day for chronic use.

## Niacinamide

In mice studies, niacinamide was found to have properties in common with benzodiazepines and barbiturates.^64^ The vitamin was found to possess hypnotic and anticonvulsant activity, influence spinal cord activity, produce muscle relaxation and have aggression-diminishing effects. Compared with controls, patients demonstrate increased flushing, anxiety, autonomic activity, and temperature after 100 mg nicotinic acid administration, suggesting a role for nicotinic acid pathways that could be manipulated by nutritional therapies.^65^ Further controlled research is necessary to confirm and extend these pilot findings.

The DRI for niacin is 16 mg niacin equivalents/d for men and 14 mg equivalents/d for women, where 1 niacin equivalent is 1 mg niacin obtained through the diet or the metabolism of tryptophan. The term *niacin* is often used synonymously with *nicotinic acid*. Nicotinamide, the amide form of nicotinic acid, is a building block for both nicotinamide adenine dinucleotide (NAD) and nicotinamide adenine dinucleotide phosphate (NADP).

“It is supposed that the favorable therapeutic effects of nicotinamide, nicotinic acid and their active biological form—NAD—are realized due to the mechanisms of their functioning in the nervous system, for treating schizophrenia, epilepsy and other diseases of the nervous system”.^66^ Hoffer^67^ and Pauling^68^ reviewed literature pertaining to the use of various forms of niacin to treat schizophrenia. They found several studies in which success was reported with niacin therapy. However, these conclusions have been criticized^69^ for failure of the investigators to support their claims with evidence from double-blind and placebo-controlled studies, which are necessary to ascertain the efficacy of vitamin treatment of schizophrenia.

The DRI manual enumerates various adverse effects of supplemental niacin use, but these effects are usually associated with doses of nicotinic acid of ≥1500 mg/d. It appears that nicotinamide produces fewer side effects than nicotinic acid. This difference could be due to study bias, however, if significantly fewer studies with nicotinamide have been performed.

## Vitamin B12 and Folate

Several studies have linked a folate deficiency to poor response to antidepressants. One such study was conducted where 213 outpatients with major depression were given fluoxetine for eight weeks. Those subjects found to have low folate levels were not only more severely depressed but were significantly less likely to respond to fluoxetine, which can be applied to other antidepressant treatments as well.^70^ Another comparative, placebo-controlled study was done with fluoxetine, where in addition to fluoxetine treatment 127 patients with major depressive disorder were given either a folate supplement or a placebo for ten weeks. And although it was determined that it worked better in women than in men (due to men needing a higher dose of folic acid in order to reduce homocysteine levels), 93.9% of women who received the folate showed a good response, in comparison to 61.1% of women in the placebo group.^71^

Not only can folate be used to increase antidepressant response, it can be used in combination with vitamin B12 (or by itself) to combat hyperhomocysteinemia (high homocysteine levels) and reduce depressive symptoms without the use of additional antidepressant medication. One extensive study done in Finland recruited 2,313 men (ages 42–60) in between 1984 and 1989, and recorded their average folate intake until the year 2000. Those that were below the average intake level (256 mcg/day) were found to have a significantly higher risk of severe depression than those above the median level.^72^ Several other studies show that many patients diagnosed with depression have significantly lower folate levels than controls.^73^ Both folate and B12 deficiencies are marked by high levels of homocysteine, which are increased among depressed patients.^74^

Thirty percent of patients hospitalized for depression were deficient in B12,^75^ and people who are deficient in B12 were 2.05 times more likely to be severely depressed than non-deficient subjects (28).^76^ One third of depressed patients had low folic acid levels, with treatment improving their depression.^77^ Low serum folate and B12 levels predicted refractory responses to antidepressant medication.^78^,^79^,^80^,^81^ The DRI for vitamin B-12 is 2.4 μg/d for adults. Cobalamin is the precursor to methylcobalamin and adenosylcobalamin, the bioactive cofactor forms of cobalamin. The DRI for folic acid is 400 μg/d.

### Methylenetetrahydrofolate reductase (NADPH), schizophrenia, rages, and depression

MTHFR catalyzes the conversion of 5,10-methylenetetrahydrofolate to 5-methyltetrahydrofolate. The latter is the predominant circulatory form of folate and the main carbon donor for the remethylation of homocysteine to methionine. Patients with severe MTHFR deficiency (0–20% residual activity) present in infancy or adolescence with developmental delay, motor and gait dysfunction, seizures, schizophrenic disturbances, and other neurologic abnormalities; they are also at risk of vascular complications. MTHFR mutations, including the 677C → T polymorphism, lead to elevated plasma homocysteine concentrations, a risk factor for vascular disease and possibly schizophrenia.^82^

Two children who were found to have homocystinuria after they were examined for rages and seizures were initially thought to have a biochemical defect in the conversion of homocysteine to methionine. They both responded favorably to low-dose folic acid (0.8–3 mg/d) with a decrease in urinary homocystine and other improvements, but the benefits only lasted several months, at which time homocystine concentrations increased and rages resumed. In one case, double the amount of folate and a low-protein diet caused improvement; in the other, betaine and a low-protein diet were effective. The authors suggested that the biochemical defect was a deficient MTHFR activity that is partially responsive to folate therapy.^83^

Another reason for lowering homocysteine concentrations by riboflavin, vitamin B-12, folate, and vitamin B-6 supplementation is the reduction of anger and hostility. Positive and significant associations were reported between hostility and homocysteine concentrations in both men and women and between anger and homocysteine concentrations in men.^84^

The 677C → T polymorphism in *MTHFR* was examined in persons with schizophrenia, major depression, and bipolar disorder.^85^ The *TT* variant was found in 12% of 419 control subjects, 21% of 297 patients with schizophrenia (*p* < 0.0006; *p* < 0.002 after Bonferroni correction), 28% of 32 patients with major depression (*p* < 0.06; *p* < 0.02 after Bonferroni correction), and 13% of 40 patients with bipolar disorder (NS). The authors pointed out that the oxidation product of homocysteine, homocysteic acid, exerts potent excitatory effects. An overrepresentation of the *TT* variant has been found in people with schizophrenia who responded to neuroleptics compared with that in control subjects.^86^

In another study, high homocysteine concentrations were found in 9 of 20 patients with schizophrenia.^87^ The thermolabile 677C → T polymorphism was screened for in a follow-up study of 11 patients with high homocysteine concentrations. Seven of the 11 patients, 6 males and 1 female, had the homozygous *TT* genotype. One male patient was heterozygous and all 3 normal homozygotes were females. In the patients who were homozygous for the polymorphism, homocysteine concentrations did not respond to vitamin B-12 but were normalized by folate supplementation. In the healthy homozygotes, however, homocysteine concentrations were reduced by vitamin B-12 alone. It was concluded that homozygosity for thermolabile MTHFR may be a risk factor for schizophrenia-like psychosis, and that this risk might be reduced by folate supplementation.^88^ In a small study, homocysteine was significantly higher in patients with schizophrenia who had low serum folate concentrations (*n* = 6) than in control subjects with low serum folate concentrations (*n* = 8).^89^

Clinical trials of B vitamin therapy (including folate and riboflavin) in relation to schizophrenia and rages are warranted on the basis of the association of higher homocysteine concentrations with anger^90^ and schizophrenia^91^ and the association of the *TT* genotype with schizophrenia. Homocysteine accumulation can be an indicator of a defective enzyme in the methylation pathway and treatment with vitamin precursors of substrates and cofactors in that pathway such as riboflavin, vitamin B-12, folate, and vitamin B-6 may be beneficial in managing rages and schizophrenia. A UL for folate intake from supplements and fortified foods has been set at 1000 μg/d for adults and 300 μg/d for 2-y-olds increasing to 800 μg/d for 16-y-olds, although higher amounts seem warranted in many cases.

## Vitamins C and E

A case control study was done in 2004 that looked at 93 institutionalized subjects (15 of which had Alzheimer’s disease and 28 of which had senile dementia) and 50 subjects who were mentally healthy, all of whom were aged 65+ years. Despite having similar dietary intakes of vitamin C, the average plasma vitamin C levels were much higher in the control group than those who had some form or dementia (0.84 mg/dl versus 0.56 mg/dl).^92^ However, vitamin C supplementation has proven more effective when taken with vitamin E. As vitamin C is oxidized, it appears to regenerate the vitamin E, acting as an antioxidant. This in turn promotes the oxidation of vitamin E and its ability to deactivate other free radicals.^93^

The Honolulu-Asia Aging Study is a perfect example of this vitamin C–E synergy in which 3,385 Japanese-American men, ages 71–93, were investigated, 386 of which had some kind of dementia, Alzheimer’s, or showed cognitive difficulties. This data was obtained in 1982, in addition to information about their supplement intake/use. They were questioned again in 1988, and were finally assessed for dementia and mental abilities between 1991 and 1993. Those taking vitamins C and E on a regular basis in 1988 were 88% less likely to have vascular dementia four years later. Of those in 1991–93 who still did not have any form of dementia, the men that took vitamins C and E supplements from 1988 were 20% more likely to have better cognitive function, and those who took the supplements in 1982 and 1988 had a 75% greater chance of better mental performance.^94^

A similar study was done from 1995 to 2000, where 4,740 men and women (ages 65 and older) were assessed in 1995–97 and again in 1998–2000. The initial visit found 200 prevalent cases of Alzheimer’s, whereas the follow-up visit found 104 new/incident cases. The greatest reduction in prevalence and incidence was found in those who took vitamins C and E supplements in combination with each other, about 78% in prevalence and about 64% in incidence.^95^ Even more interesting is vitamin E’s abilities to not only help prevent Alzheimer’s, but slow the progression of the disease as well; 341 patients with the disease (of about moderate severity) were treated with 2000 units a day of vitamin E. When compared to a placebo group, the patients treated with vitamin E showed significantly less progression of the disease at the end of the two year course treatment.^96^ Alpha-Tocopherol is the main form of the lipid-soluble vitamin E in animal tissues and plasma. The DRI for vitamin E is 15 mg/d (× 1.5 IU/mg = 22.5 IU/d) as α-tocopherol.^97^ The UL of vitamin E on the basis of supplementation with α-tocopherol is 1000 mg/d (1500 IU) as a result of adverse effects including increased risk of hemorrhage.^98^ In 2 patients mentioned above, the reinstatement of α-tocopherol treatment showed a linear relation to serum α-tocopherol concentrations. The maximal dosage (40 mg•kg body wt^−1^•d^−1^) resulted in a plasma concentration >50 μmol/L.^99^

DHEA (dihydroepiandosterone) supplementation has been shown beneficial for major depression, especially for women over age 70.^100^ S-Adenosylmethionine (SAMe) is a substance found naturally in the human body which serves as a methyl donor in many synthetic reactions, and may contribute to an increase in the levels of certain neurotransmitters when given in supplement form. It appears to have enough of an antidepressant effect to warrant further research.^101^,^102^,^103^ Other B vitamins have been examined for a role in depression as well, in particular vitamin B6 and folic acid.^104^,^105^,^106^,^107^, ^108^,^109^ One third of depressed patients have low folic acid levels, with treatment improving their depression.^110^ Low serum folate and B12 levels predict refactory responses to antidepressant medication.^111^,^112^,^113^,^114^ Inositol is, unofficially, a B vitamin that is found in cell membranes (phosphatidylinositol) where it functions closely with choline.^115^ Inositol was equivalent to single-drug therapy for depression and panic disorder in one study and is a potential antidepressant.^116^,^117^ One study found that 75% of depressed patients were magnesium deficient with another 9% at borderline levels.^118^ Magnesium supplementation was helpful.^119^ Melatonin has been reported effective with 84% of depressed patients showing abnormal levels.^120^

Amino acid supplementation has been reported helpful in some patients with depression.^121^ Tryptophan has long been reported effective for depression, often in combination with vitamins B3 and B6.^122^,^123^

## Conclusion

These are just a few examples of how micronutrients can play a beneficial role in the treatment of several psychiatric illnesses. As researchers continue to investigate the efficacy of nutritional healing, more and more information will be readily available to those wishing to take a more natural course of therapy. For the time being, we can rely on the already thousands of successful studies that “alternative therapies” need not be an alternative anymore.
